# Piperacillin/tazobactam‐associated myoclonic status epilepticus in a patient with end‐stage renal failure on hemodialysis

**DOI:** 10.1002/epd2.70302

**Published:** 2026-05-30

**Authors:** O. M. Malanga, G. Giovannelli, G. Pastorelli, S. Fittante, A. Poggesi, E. Rosati

**Affiliations:** ^1^ Department of Neuroscience, Psychology, Drug Area and Child Health (NEUROFARBA) University of Florence Florence Italy; ^2^ Emergency Neurology, Department of Neuroscience and Sense Organs Careggi University Hospital Florence Italy

**Keywords:** beta‐lactams neurotoxicity, hemodialysis, myoclonic status epilepticus, piperacillin/tazobactam, seizures

Neurotoxicity related to beta‐lactam antibiotics is an uncommon but well‐recognized complication, with manifestations including encephalopathy, myoclonus, seizures, and status epilepticus, most often observed in patients with multiple comorbidities, particularly end‐stage renal disease.^1^, ^2^, ^3^ Among this class of drugs, piperacillin/tazobactam has been infrequently linked to seizures and non‐convulsive status epilepticus (NCSE), mostly with concomitant encephalopathy.^4^, ^5^, ^6^, ^7^ We describe the case of an elderly woman with end‐stage renal disease on hemodialysis who developed an electroclinical myoclonic status epilepticus during piperacillin/tazobactam therapy.

A 75‐year‐old woman without a history of epilepsy was hospitalized for endovascular revascularization because of severe peripheral arterial disease of the left lower limb. Her medical history included heart failure with preserved ejection fraction, end‐stage chronic kidney disease on maintenance hemodialysis, arterial hypertension, type 2 diabetes mellitus complicated by left diabetic foot, and an ischemic lacunar stroke in 2011. At the time of admission, her home medications included amlodipine, atorvastatin, aspirin, clopidogrel, bisoprolol, gabapentin, tapentadol, and linagliptin.

Because of necrosis involving the first, third, and fourth toes of the left foot, she had initially been started on amoxicillin/clavulanate at home a few days before admission. On hospitalization, antibiotic therapy was switched to piperacillin/tazobactam, with dose adjustment according to renal function (2.25 g every 8 h); a scheduled high‐flux hemodialysis session was performed on the second day after admission. On day 5 of piperacillin/tazobactam treatment, the patient developed subcontinuous myoclonic jerks involving the limbs and the cranial district, prompting neurological evaluation.

At initial assessment, she exhibited subcontinuous myoclonic movements involving the limbs, face, and tongue, with brief interruptions of speech associated with the jerks that made her unable to communicate verbally but still able to cooperate during the examination. The EEG (Figure 1A,B) showed subcontinuous generalized paroxysm of polyspike and spike‐and‐wave complexes, with maximal amplitude over the frontocentral regions and time‐locked to the activity as recorded on polygraphic channels, consistent with myoclonic status epilepticus. No major electrolyte, glucose, BUN, or ammonia abnormalities were documented. Brain CT showed no signs of an acute intracranial pathology, with only chronic microvascular ischemic changes and bilateral nucleo‐capsular lacunar sequelae.

**FIGURE 1:**
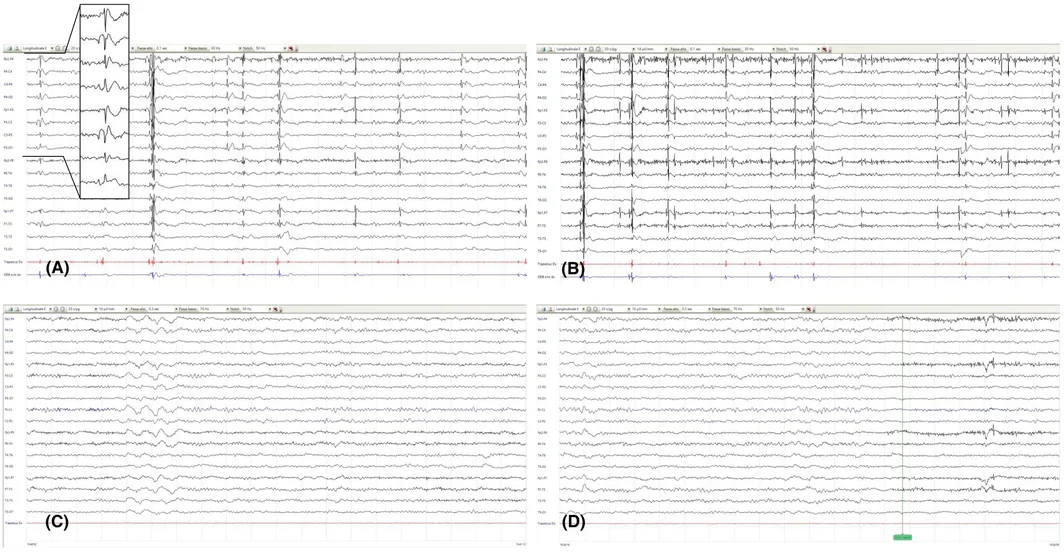
Standard EEG recordings (10–20 system, longitudinal montage, epoch duration 20 s. (A, B) High‐pass filter 0.1 s, low‐pass filter 30 Hz, gain 14 μV/mm; (C, D) High‐pass filter 0.3 s, low‐pass filter 70 Hz, gain 10 μV/mm). (A–B) Abundant generalized spike‐ and polyspike–wave paroxysms time‐locked to myoclonic jerks recorded on EMG channels from the right trapezius and orbicularis oris, supporting the diagnosis of myoclonic status epilepticus. Panel A includes a magnified inset of a spike–wave element. (C, D) No epileptiform paroxysms or EMG activity were observed on the EEG performed after the antiseizure therapy, with only infrequent bilateral frontally predominant delta waves and a modest eye‐opening reactivity.

The patient was initially treated with repeated intravenous boluses of diazepam (total dose 10 mg), followed by a loading dose of levetiracetam 500 mg, with prompt resolution of the myoclonic jerks and improvement in cognition and verbal fluency. Piperacillin/tazobactam was promptly discontinued, and the scheduled high‐flux hemodialysis session was anticipated. Maintenance treatment with levetiracetam 250 mg twice daily was started.

The follow‐up EEG (Figure 1C,D) performed the next day did not show any epileptiform discharges, with a slowing of the background activity and infrequent bilateral frontally predominant delta waves.

At the 3‐month follow‐up visit, the patient reported no further involuntary movements or episodes of impaired awareness. Given the persistence of major predisposing factors, including end‐stage renal disease on hemodialysis and the likelihood of future antibiotic exposure because of ongoing infectious risk, continuation of levetiracetam was considered appropriate.

This case broadens the clinical spectrum of piperacillin/tazobactam‐related neurotoxicity. Most previously reported cases have emphasized an encephalopathic presentation, sometimes associated with seizures, whereas our patient showed prominent generalized myoclonus accompanied by only transient impairments of consciousness associated with the jerks.

The pathophysiological basis of beta‐lactam neurotoxicity is thought to depend mainly on impaired inhibitory neurotransmission, particularly through interference with GABA‐A‐mediated activity, leading to increased neuronal excitability. In patients with renal dysfunction, reduced drug clearance may promote antibiotic accumulation and further increase the risk of neurotoxicity.^1^, ^2^

Alternative or contributory explanations should be carefully considered in these settings. End‐stage renal disease itself may predispose to myoclonus and encephalopathy, while intercurrent infection, metabolic disturbances, and accumulation of concomitant renally excreted medications may all contribute to neurological worsening.^8^, ^9^ In our patient, some of these factors were relevant; however, the close temporal relationship between treatment initiation and symptom occurrence, the absence of major metabolic abnormalities sufficient to explain the entire picture, and the prompt improvement after diazepam administration and withdrawal of the antibiotic suggested a major pathogenic role of the piperacillin/tazobactam.

From a practical perspective, in dialysis patients and, more broadly, in patients with multiple comorbidities receiving beta‐lactam antibiotics, the onset of myoclonus with or without fluctuating consciousness should prompt urgent EEG evaluation as such symptoms can reflect an underrecognized and treatable electroclinical status epilepticus.

## FUNDING INFORMATION

No funding was received.

## CONFLICT OF INTEREST STATEMENT

The authors declare that there are no conflicts of interest related to this work.

## CONSENT

Informed consent was obtained from the patient.

**Test yourself**

1. What is the proposed mechanism underlying beta‐lactam–related neurotoxicity?
  - Dopaminergic blockade in the basal ganglia.
  - Interference with GABA‐A‐mediated inhibitory neurotransmission.
  - Autoimmune antibody production against neuronal surface antigens.
  - Direct sodium channel blockade.
  - Excess glutamatergic excitatory neurotransmission.
2. Which statement regarding beta‐lactam‐related neurotoxicity is incorrect?
  - It may present with encephalopathy, myoclonus, or seizures.
  - It is more likely to occur in patients with impaired renal function.
  - It is typically unrelated to drug accumulation.
  - It may manifest as electroclinical status epilepticus.
  - It can improve after withdrawal of the offending drug.
3. Which of the following conditions is least likely to account for new‐onset myoclonus in this clinical setting?
  - Uremia‐related metabolic encephalopathy.
  - Sepsis‐associated encephalopathy.
  - Accumulation of renally excreted medications.
  - Chronic microvascular ischemic changes.
  - Antibiotic‐related neurotoxicity.

*Answers may be found in the* *supporting information*.

## Supporting information


Data S1.

## Data Availability

Data sharing is not applicable to this article as no new data were created or analyzed in this study.
