# Supplementary material for: Piperacillin/tazobactam‐associated myoclonic status epilepticus in a patient with end‐stage renal failure on hemodialysis
Source: Epileptic Disord. 2026 May 30;28(5):1701–4. doi: 10.1002/epd2.70302 (PMC13695470; doi:10.1002/epd2.70302)
Supplement: Supplementary file 1 [file EPD2-28-1701-s001.docx]

**Answers**

**1. Correct answer: B.**

**Beta-lactam antibiotics are known to interfere with GABA-A-mediated inhibitory neurotransmission, leading to increased neuronal excitability and seizures or status epilepticus. The other options describe mechanisms not typically involved in beta-lactam neurotoxicity.**

2. **Correct answer:** C.

Beta-lactam neurotoxicity is often related to drug accumulation, particularly in patients with renal impairment. It commonly presents with encephalopathy, myoclonus, seizures, or status epilepticus, and typically improves after discontinuation of the causative drug.

3. Correct Answer: D.

Chronic microvascular ischemic changes are common in elderly patients but are unlikely to explain acute-onset myoclonus. The other options represent recognized causes of myoclonus in patients with advanced renal disease.
